# Thermodynamics of Heat Pump and Refrigeration Cycles

**DOI:** 10.3390/e23050524

**Published:** 2021-04-25

**Authors:** Alojz Poredoš

**Affiliations:** Faculty of Mechanical Engineering, University of Ljubljana, 1000 Ljubljana, Slovenia; alojz.poredos@fs.uni-lj.si

Energy consumption for heating and cooling in buildings and industry accounts for almost half of total energy consumption in all sectors [1]. Therefore, the heating and cooling sector has a huge potential to reduce primary energy consumption and achieve sustainable development objectives.

While energy use for heating decreased significantly in the last decade due to a number of energy efficiency-related measures, energy use for cooling is increasing significantly. Simultaneous or separate needs for heating and cooling energy can be satisfied with the use of a heat pump. Such a solution is undoubtedly very energy efficient and economically successful. Appropriate thermodynamics of refrigeration cycles are essential for efficient operation of a heat pump. 

All published articles have a common exergy approach to the analyses of thermodynamic processes. This indicates the authors’ special concern for the minimum use of primary energy for heating and cooling. 

Authors of the paper “Exergy Analysis of Fluidized Desiccant Cooling System” [2] analyzed the exergy destruction in all components of a fluidized desiccant cooling system. They have shown that the main sources of exergy destruction are in fluidized beds and regenerative heat exchangers and indicated the possibilities for improving exergy efficiency of the whole process. 

In the paper “Exergetic and Economic Evaluation of a Transcritical Heat-Driven Compression Refrigeration System with CO_2_ as the Working Fluid under Hot Climatic Conditions” [3], the authors devoted special attention to minimum exergy destruction and to cost optimization at various operating conditions. They showed a significant increase in exergy efficiency in the case when the system operates in cogeneration mode if the available heating capacity within the gas cooler is utilized. 

A combination of a vapor compression refrigeration system with a vapor absorption refrigeration system is described and analyzed in the paper “Superstructure-Based Optimization of Vapor Compression-Absorption Cascade Refrigeration Systems” [4]. The authors used a superstructure-based optimization model to find the most energy-efficient and cost-effective configuration of the compression–absorption cascade refrigeration system. 

The new fundamental step in the direction of upper bounds for the COP of the Carnot irreversible refrigerator can be found in the paper: “Effect of Machine Entropy Production on the Optimal Performance of a Refrigerator”. The main contribution of the authors to more energy-efficient refrigeration processes is a novel and more complete modelling of an irreversible Carnot refrigerator based on heat transfer entropy, which involves the coupling between sink (source) and machine through a heat transfer constraint [5].

The paper “Exergy Analysis of Advanced Adsorption Cooling Cycles” gives us novel results of an advanced adsorption cooling cycle in different operational regimes. The authors determined exergy flows and its destruction in all phases of the cooling cycle by careful exergy analysis. They found that the highest exergy efficiency of the entire cooling cycle was achieved in the case of combined heat and mass advanced recovery cycle [6].

Results of performance and exergy analyses of a complex heating system based on a heat pump with different heat sources are presented in the paper “Performance and Exergy Analyses of a Solar Assisted Heat Pump with Seasonal Heat Storage and Grey Water Heat Recovery Unit”. The detailed exergy analysis and comparison of annually averaged exergy efficiencies of different heat sources in space heating operation mood, shows the best results in the case of a system based on a water–water heat pump, assisted with solar thermal collectors and seasonal heat storage [7]. 
